# Maternal High-Risk Fertility Behavior and Its Associated Factors in Hadiya Zone, Southern Ethiopia: A Facility-Based Cross-Sectional Study

**DOI:** 10.4314/ejhs.v34i2.2

**Published:** 2024-03

**Authors:** Samuel Kusheta, Robel Demelash, Elias Kenea, Genet Kasa, Woineshet Ermako, Haregewoin Limenih, Wudu Yesuf

**Affiliations:** 1 Department of Public Health, Hossana College of Health Sciences, Hossana, Ethiopia; 2 Department of Emergency Medical Technology, Hossana College of Health Sciences, Hossana, Ethiopia; 3 Department of Reproductive Health, Faculty of Health Sciences, Wachemo University, Hossana, Ethiopia; 4 Department of Public Health, Mizan-Aman College of Health Sciences, Aman, Ethiopia

**Keywords:** High-risk fertility, associated factors, Ethiopia

## Abstract

**Background:**

The fertility behavior of women is characterized by maternal age, birth spacing and order, and it impacts the health of women and children. Evidence on the factors associated with maternal high-risk fertility behavior is scant in Ethiopia. Thus, the aim of this study was to identify the factors associated with maternal high-risk fertility behavior in Hadiya Zone, Southern Ethiopia.

**Methods:**

A facility-based cross-sectional study was conducted. Three hundred women of reproductive age admissions to public hospitals in the Hadiya Zone who gave birth in the five years preceding this study were selected using systematic random sampling. Face-to-face interviews were held to gather data using interviewer-administered questionnaires. Descriptive statistics and binary logistic regression models were used to analyze data. Statistical significance was assessed using odds ratios and 95% confidence intervals and declared at a p-value of less than 0.05.

**Results:**

The overall proportion of maternal high-risk fertility behavior was 60.3%. Mother, who lived in rural areas (AOR = 4.85; 95% CI: 2.56, 9.19), had early marriage (AOR = 3.39; 95% CI: 1.87, 6.14) and had unplanned last pregnancy (AOR = 2.62; 95% CI: 1.28, 5.39) were more likely engaged in high-risk fertility patterns.

**Conclusions:**

In the study area, there was a high overall proportion of married women engaging in high-risk fertility behavior. Mothers with early marriages, unplanned pregnancies, and rural residence were more likely engaged in high-risk fertility behaviors. Plans for interventions aimed at preventing maternal high-risk fertility behavior should center on expanding access to family planning services and ending the practice of early marriage by giving rural women extra care and attention.

## Introduction

The fertility behavior of women is characterized by maternal age, birth spacing and order, that have an effect on both the health of the mother and that of the child (1,2). Maternal high-risk fertility behaviors generally include too early or too late childbearing, a higher number of live births, and short birth intervals (3).

By 2030, the Sustainable Development Goals (SDGs) of the United Nations (UN) calls for a maternal mortality ratio of less than 70 per 100,000 live births and an under-five mortality rate of 25 per 1000 live births (4). With the current trend of reduction, Ethiopia will be under challenge in achieving the target as of the deadline, since the under-five mortality rate was 67 deaths per 1,000 live births, and maternal mortality ratio was 412 per 100,000 live births in 2019 (5). High-risk fertility behavior has an adverse influence on maternal and child health (6, 7), which hampered efforts to reduce maternal and child morbidity and mortality in Ethiopia (6). Despite effective interventions such as increasing health services accessibility and coverage, providing maternal health services and postnatal care follow-up free of charge (8), maternal risky fertility behavior remains highly prevalent and become a major public health concern in Ethiopia (6). Overall, 77% of married women in Ethiopia have the potential for having high-risk birth; 31% of these women fall into a single high-risk category, and 45% of them fall into multiple high-risk categories (5).

Identifying the risk factors for maternal high-risk fertility behavior will also play a critical role in curbing childhood and maternal mortality (3). Three-fifths (62%) of births in Ethiopia are associated with an elevated risk of maternal death from avoidable risks, 38% of births are in a single high-risk category, and 24% of births are in a multiple high-risk category (5). The chance of morbidity and mortality of under-five children was high among children born to mothers having high-risk fertility behaviors in Southern Ethiopia (7, 9).

Some studies documented that social factor like education, marital status, household wealth index, and place of residence contribute to the prevalence of high-risk pregnancies (6, 10, 11). Unwanted pregnancies resulting from imprudent planning often create high-risk fertility among married and unmarried women (10, 12). Evidence of the factors associated with maternal high-risk fertility behavior was scant in Ethiopia, and the limited study findings were based on the Ethiopian demographic and health survey data. Therefore, this study aimed to identify the factors associated with maternal high-risk fertility behavior in the Hadiya Zone of Southern Ethiopia based on primary data.

## Materials and Methods

**Study design and period**: A facility-based cross-sectional study was conducted. The study was conducted from April 01 to June 30, 2021.

**Study setting**: All four of the hospitals that are currently in operation in the Hadiya zone – the Nigist Eleni Mohammed Memorial Comprehensive Hospital, and Homecho, Gimbichu, and Shone primary hospitals were used for this study. In 2018, there were 1,650,104 people living in the Hadiya Zone (820,102 males, 830,002 females, and 384,474 women of childbearing age). There are four town administrations and 13 districts in the zone. Situated 230 kilometers southwest of Ethiopia's capital, Addis Ababa, Hossana serves as the capital town of the Hadiya Zone, which is home to the referral hospital. The three district hospitals are situated in semi-urban areas. Rural health centers serve as the referral hub for district hospitals and referral hospitals. Comprehensive emergency obstetric care services are offered by all hospitals. In 2022, there were 57,094 live births in the Hadiya Zone (7, 13).

**Participants**: Childbearing-age women who gave birth in the five years preceding this study and who were admitted to public hospitals in the Hadiya zone of Southern Ethiopia were the study participants.

**Inclusion and exclusion criteria**: Women of reproductive age (15-49 years) admissions to study hospitals who had gone through childbirth in the five years preceding this study and whose children were alive or died were included. Participants with permanent difficulty in communication physiologically or in a state of not able to respond to the interview were excluded from the study.

**Sample size and sampling procedure**: With the use of Epi-Info version 7.2.2.6 software, the sample size for the study was estimated for a cross-sectional study and single population survey. The assumptions included a 95% confidence level, 80% power, population size > 10,000, 76.9% expected frequency or proportion of reproductive-age women who had at least one high-risk fertility behavior, and odds ratios for significant determinant factors (unwanted pregnancy, education) derived from an analysis of 2016 Ethiopian demographic and health survey data (14), a 1:1 exposed to non-exposed ratio, and an acceptable margin of error of 5%. Thus, the final minimum sample size included in the study was 273 + 10% non-response rate = 300, since the sample size computed for a single population survey produced the largest sample size.

**Figure 1:**
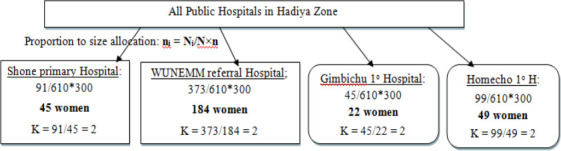
Schematic diagram showing sampling procedure, i.e. proportional allocation of sample size to public hospitals in Hadiya Zone, 2021

The sample size was distributed proportionately to each of the study hospitals using, the last three months' worth of maternity admissions reports from each public hospital. Systematic random sampling was used to select women of childbearing age admitted to public hospitals, and the maternity admissions of the individual hospitals were used to calculate the K^th^ interval (Figure 1).

### Study variables

**Outcome variable**: The outcome variable was maternal high-risk fertility behavior (Non-risk, High-risk category). The study examined maternal high-risk fertility behaviors in relation to birth interval, birth order, and mother's age at delivery (3). To be more precise, any of the following conditions were classified as high-risk fertility behavior (coded as 1 and otherwise 0): mother is less than 18 years old at the time of delivery; mother is older than 34 years old; the most recent child was born less than 24 months after the previous child; and the most recent child in the birth order is older than 3.

Any high-risk versus non-risk fertility behavior was coded as 1/0, respectively, for a single high-risk category in order to facilitate further analysis. On the other hand, combinations of two or more conditions as a composite variable were treated as multiple high-risk categories as specified.

**Exposure variables**: Socio-demographic factors (age, residence, educational level, maternal occupation, husband occupation, husband education, monthly income, marital status, and media exposure) and reproductive health characteristics (contraceptive use, wanted pregnancy, antenatal care follow-up, stillbirth, place of delivery, and women autonomy) were the exposure variables.

In order to calculate the women's autonomy index, questions concerning who in the household makes decisions about major household purchases, visiting family and relatives, maternal healthcare, and child health care were posed. A respondent received one point for each of the four questions if she participated in the decision, and zero points if not. The sum of these points produced total scores ranging from 0 to 4 (7, 15). The score for each respondent was calculated by dividing it by the highest possible score of 4. A score of more than 0.5 was regarded as autonomous. By adding up each respondent's score and dividing the total by the number of responses, the average value of the women's autonomy index was determined (15).

**Data collection procedure and instrument**: Data were collected using a structured interviewer-administered questionnaire which was developed after a thorough review of the literature (1-3,10-12,14-16). Face-to-face interviews were held to gather data. For the purpose of gathering data, the study hospitals' maternity wards and clinics were visited. Data was gathered through a retrospective birth history survey, wherein women participants listed all of the children they had ever had, along with the dates of birth of each child.

**Data quality control**: To ensure consistency, the English questions were translated into Amharic and then back into English by different professional translators. For one week, using 5% of the sample size, the pre-test was conducted at Worabe Comprehensive Specialized Hospital. During that time, any inconsistencies in the tool were fixed. The data collectors received two days of training on the objectives of the study, data collection methods, and tools. A trained supervisor and the principal investigator also regularly verified the consistency and completeness of the data, making any necessary corrections on the spot. Each questionnaire was coded after the data was gathered, and data cleaning was done before the data analysis process began.

**Data analysis**: Each questionnaire was checked for completeness, coded, and entered into Epidata Version 4.4 and exported to the Statistical Package for Social Sciences for Windows version 24 for analysis. The study population was described using frequencies, proportions, and measures of variation with regard to socio-demographic and other pertinent variables. A binary logistic regression model was constructed using a backward likelihood ratio.

The association between the independent variables and the outcome variable was examined using bivariate logistic regression, and variables for the final multivariable logistic regression model were selected based on a p-value of less than 0.25. Using the Hosmer and Lemeshow goodness of fit test, we evaluated the final model's fitness and determined that it was fit (X^2^ = 5.18, p-value = 0.738). Odds ratios and 95% confidence intervals were used to evaluate statistical significance, which was declared at a p-value of less than 0.05.

**Ethics approval**: The study received ethical approval on April 01, 2021, from Hossana College of Health Sciences, institution review board with the reference number ‘HCHS-5030’ and ‘Code 1001/2013 EC’.

The relevant authorities at each public hospital in the Hadiya Zone gave their approval. Additionally, after explaining the objective and purpose of the study to each mother and giving them information about their right to withdraw from the study at any time, each mother gave informed consent. Information confidentiality was guaranteed, and de-identified and de-linked data were kept in a safe place.

## Results

**Socio-demographic characteristics**: With a 100% response rate, 300 study participants in total were included in the analysis. The mean age of participant mothers was 30.1 years ± 4.7. Two-thirds of the mothers who participated were from rural areas (64.3%), and the majority of them were between the ages of 25 and 30 years (45.7%). More than half of the husbands (54.0%) and half of the mothers (50.3%) completed primary school.

The percentage of mothers who engage in high-risk fertility behavior declines as education levels rise (p-value < 0.001). Nearly half of the participant mothers' husbands (49.0%) were farmers, while three-fourths of the mothers (78.0%) worked as housewives. The family's estimated monthly median income was $73.5 (IQR $56.7-$105.0). Ninety percent of the participants were exposed to the media, with radio being the most common medium (Table 1).

**Table 1:** Socio-demographic characteristics of mothers admitted to public hospitals in Hadiya Zone, Southern Ethiopia (n = 300)

Variables	Category	Frequency (percentage)		Total

		HRFB	No HRFB	
Mother's age in years	19-24	4 (11.1%)	32 (88.9%)	36 (12.0%)
Range (19-40)	25-30	54 (39.4%)	83 (60.6%)	137 (45.7%)
	31-36	84 (97.7%)	2 (2.3%)	86 (28.7%)
	37-40	39 (95.1%)	2 (4.9%)	41 (13.7%)
Residence	Urban	33 (30.8%)	74 (69.2%)	107 (35.7%)
	Rural	148 (76.7%)	45 (23.3%)	193 (64.3%)
Mother's educational status	No formal education	58 (95.1%)	3 (4.9%)	61 (20.3%)
	Primary	86 (57.0%)	65 (43.0%)	151 (50.3%)
	Secondary	27 (42.9%)	36 (57.1%)	63 (21.0%)
	Higher education	10 (40.0%)	15 (60.0%)	25 (8.4%)
Husband's educational status	No formal education	43 (82.7%)	9 (17.3%)	52 (17.3%)
	Primary	95 (58.6%)	67 (41.4%)	162 (54.0%)
	Secondary	28 (66.7%)	14 (33.3%)	42 (14.0%)
	Higher education	15 (34.1%)	29 (65.9%)	44 (14.7%)
Mother's occupation	Housewife	147 (62.8%)	87 (37.2%)	234 (78.0%)
	Merchant	13 (46.4%)	15 (53.6%)	28 (9.3%)
	Civil servant	21 (55.3%)	17 (44.7%)	38 (12.7%)
Husband's occupation	Farmer	110 (74.8%)	37 (25.2%)	147 (49.0%)
	Merchant	43 (53.8%)	37 (46.3%)	80 (26.7%)
	Civil servant	17 (37.8%)	28 (62.2%)	45 (15.0%)
	Driver	11 (39.3%)	17 (60.7%)	28 (9.3%)
The average estimated monthly income of the family in USD	$21.0-100.9	136 (64.8%)	74 (35.2%)	210 (70.0%)
	$101.0-180.9	15 (30.0%)	35 (70.0%)	50 (16.7%)
	$181.0-260.9	30 (75.0%)	10 (25.0%)	40 (13.3%)
Media exposure	Yes	161 (59.6%)	109 (40.4%)	270 (90.0%)
	No	20 (66.7%)	10 (33.3%)	30 (10.0%)

HRFB: High-Risk Fertility Behavior; USD: **United States Dollar; 1 Ethiopian birr equals 0.021 USD**

A version of the analysis of this part was published elsewhere with different objectives (7).

**Reproductive health characteristics**: Participants in the study had families ranging in size from 3 to 10, with a mean size of 5.7 ± 1.9. The median age of mothers at marriage was 19 (IQR 18-20). The majority of participant mothers gave their last birth at a health facility (87.7%), and 90% of them had antenatal care (ANC) follow-up for their last pregnancy. The proportion of mothers engaging in high-risk fertility behavior significantly declines as the number of ANC contacts increases (p-value = 0.033), with two-thirds of mothers having 1-3 ANC contacts for their most recent delivery, with a median of 3 (IQR 3-4). Sixty-one percent of mothers used contraceptives prior to their most recent pregnancy, and 86.0% of mothers had ever given stillbirth. Of the participating mothers, 35.3% had received postnatal care (PNC) follow-up after their most recent delivery, and 56.7% had planned and desired their most recent pregnancy.

Maternal high-risk fertility behaviors were found to be statistically significantly associated with both the type of unplanned pregnancy and the absence of PNC (p-value < 0.001). The women's autonomy index had an average value of 0.71, and 54.1% of the mothers who took part in the study were autonomous (Table 2).

**Table 2:** Reproductive health characteristics of participant mothers in Hadiya Zone public hospitals, Southern Ethiopia (n = 300)

Variables	Category	Frequency (percentage)		Total

		HRFB	No HRFB	
Family size	3-6	69 (37.3%)	116 (62.7%)	185 (61.7%)
	7-10	112 (97.4%)	3 (2.6%)	115 (38.3%)
Maternal age at marriage	14-19 years	135 (73.4%)	49 (26.6%)	184 (61.3%)
	20-25 years	46 (39.7%)	70 (60.3%)	116 (38.7%)
Place of last delivery	Home	25 (67.6%)	12 (32.4%)	37 (12.3%)
	Health facility	156 (59.3%)	107 (40.7%)	263 (87.7%)
ANC for the last delivery	Yes	159 (58.9%)	111 (41.1%)	270 (90.0%)
	No	22 (73.3%)	8 (26.7%)	30 (10.0%)
Number of ANC contacts (n = 270)	1-3 contacts	109 (63.7%)	62 (36.3%)	171 (63.3%)
	> 4 contacts	50 (50.5%)	49 (49.5%)	99 (36.7%)
Ever gave stillbirth	Yes	30 (71.4%)	12 (28.6%)	42 (14.0%)
	No	151 (58.5%)	107 (41.5%)	258 (86.0%)
Used contraceptive before last pregnancy	Yes	116 (63.4%)	67 (36.6%)	183 (61.0%)
	No	65 (55.6%)	52 (44.4%)	117 (39.0%)
PNC for index delivery	Yes	45 (42.5%)	61 (57.5%)	106 (35.3%)
	No	136 (70.1%)	58 (29.9%)	194 (64.7%)
Type of pregnancy	Planned & wanted	79 (46.5%)	91 (53.5%)	170 (56.7%)
	Unplanned but wanted	102 (78.5%)	28 (21.5%)	130 (43.3%)
Women autonomy	Non-autonomous	97 (70.3%)	41 (29.7%)	138 (46.0%)
	Autonomous	84 (51.9%)	78 (48.1%)	162 (54.0%)

ANC: Antenatal Care; HRFB: High-Risk Fertility Behavior; PNC: Postnatal Care

A version of the analysis of this part was published elsewhere with different objectives (7)

**Maternal high-risk fertility behavior**: The mothers ranged in age from 18 to 40 at their most recent delivery, with a mean age of 30.8 +/- 4.3 years and 26.0% older than 34. The median birth interval was 3 years (IQR 2-3), and 13.4% of the mothers had< 2 years inter-birth interval. With a mean birth order of 4.1 + 1.7 and a range of 1-8, more than half (52.0%) of the children had birth orders greater than 3. Among women who were currently married, the percentage of maternal high-risk fertility behavior was 60.3%. Overall, 35.0% of these women fell into a single high-risk category, and 25.3% of them fell into multiple high-risk categories. Age >34 and birth order >3 (81.6%) were the most prevalent multiple high-risk behaviors, while birth order > 3 (77.1%) was the most common single maternal high-risk fertility behavior. Results for the profile of maternal high-risk fertility behavior were also published elsewhere as part of the project, although with different objectives (7) (Table 3).

**Table 3:** Maternal high-risk fertility behavior among currently married mothers who gave birth in the last five years, Southern Ethiopia (n = 300)

HRFB	Risk Category	Frequency	Percent
Maternal high-risk fertility behavior	Yes	181	60.3
	No	119	39.7
Type of maternal high-risk fertility behavior	Not in any high-risk category	119	39.7
	Single high-risk category	105	35.0
	Multiple high-risk categories	76	25.3
Any avoidable high-risk category			
Single high-risk category	Mother's age > 34	2	1.9
	Birth interval < 24 months	22	21.0
	Birth order > 3	81	77.1
Multiple high-risk categories	Age >34 & birth interval <24 months	1	1.3
	Age >34 & birth order >3	62	81.6
	Age >34 & birth interval <24 months & birth	13	17.1
	order >3		

HRFB: High-Risk Fertility Behavior; Mothers aged < 18 as a risk category was zero

A version of the analysis of this part was published elsewhere with different objectives (7)

**Factors associated with maternal high-risk fertility behavior**: Using bivariate binary logistic regression analysis, variables with a p-value of < 0.25 were recruited to be included in the final model. Thus, in the multi-variable analysis, the variables found to have an association with maternal high-risk fertility behavior in the final model were permanent residence, age of the mother at the time of marriage, and type of pregnancy.

Therefore, this study found that mothers residing in rural areas had 4.85 times higher odds of maternal high-risk-fertility behavior compared to urban residents (AOR = 4.85; 95% CI: 2.56, 9.19). The probability of having high-risk fertility behavior was three times higher for mothers with age at marriage 14-19 years (AOR = 3.39; 95% CI: 1.87, 6.14) compared with those 20-25 years old at the time of marriage. Moreover, those mothers with the last unplanned but wanted pregnancy type were 2.6 times more likely to experience high-risk fertility compared to those whose last pregnancy type was planned and wanted (AOR = 2.62; 95% CI: 1.28, 5.39). Antenatal care follow-up, lack of postnatal care and women's autonomy were unrelated to the probability of maternal high-risk fertility (Table 4).

**Table 4:** Multivariate logistic regression showing factors associated with maternal high-risk fertility behavior among currently married women, Southern Ethiopia, October 2021

Variables	Category	COR (95% CI)	AOR (95% CI)	P-value
Residence	Urban	1.00	1.00	
	Rural	7.37 (4.35, 12.51)	**4.85 (2.56, 9.19)**	< 0.001
Age at marriage	14-19 years	4.19 (2.56, 6.88)	**3.39 (1.87, 6.14)**	< 0.001
	20-25 years	1.00	1.00	
Number of ANC contacts	1-3	1.72 (1.04, 2.85)	1.15 (0.61, 2.17)	0.667
	>4	1.00	1.00	
PNC for the last birth	Yes	1.00	1.00	
	No	3.18 (1.94, 5.20)	1.26 (0.60, 2.62)	0.539
Pregnancy type	Planned and wanted	1.00	1.00	
	Unplanned but wanted	4.19 (2.51, 7.03)	**2.62 (1.28, 5.39)**	0.009
Women autonomy	Autonomous	1.00	1.00	
	Dependent	2.20 (1.36, 3.54)	0.91 (0.44, 1.88)	0.789
Ever gave stillbirth	Yes	1.77 (0.87, 3.62)	1.65 (0.72, 3.77)	0.233
	No	1.00	1.00	
Used contraceptive before last pregnancy	Yes	1.00	1.00	
	No	0.72 (0.45, 1.16)	0.65 (0.35, 1.20)	0.168

ANC: Antenatal Care; AOR: Adjusted Odds Ratio; CI: Confidence Interval; COR: Crude Odds Ratio; PNC: Postnatal Care

## Discussion

According to this study, among currently married women, the overall percentage of maternal high-risk fertility behavior (HRFB) was 60.3%. Of these, 35.0% fell into a single high-risk category and 25.3% into multiple high-risk categories. The demographic and health survey (DHS) data from 2016 showed that 76% of Ethiopian women of reproductive age had high-risk fertility issues (5). The prevalence of maternal high-risk fertility behavior was found to be 86.3% (16) in another study carried out in Ethiopia's Afar region, and 73.5% in another study that evaluated the spatial distribution of HRFB in Ethiopia (6). Another finding shows that 34% of Bangladeshi women had high-risk fertility patterns; of these, 28.7% engaged in a single high-risk behavior and 5.4% in multiple high-risk behaviors (3).

Our results were higher than the prevalence in Bangladesh but lower than the DHS data from Ethiopia, the study of the spatial distribution, and the study carried out in the Afar region. The mothers' socioeconomic status in the research areas may be the cause of the differences. According to one study that examined the DHS data, the Afar region is the spot area where maternal high-risk fertility behavior was highly clustered (14). Similarly, as the DHS report and the spatial distribution study constitute the spot areas, this may elevate the national prevalence compared to our study area.

This study established that mothers residing in rural areas had five times higher odds of maternal high-risk fertility behavior compared to urban residents. Similarly, women who were rural dwellers have increased odds of high-risk fertility behavior compared to those urban dwellers, according to a study conducted in Ethiopia (16). Two other studies that analyzed the Ethiopian DHS data revealed that rural dwelling was a determinant factor of maternal high-risk fertility behavior (6, 14). Another study that relied on recent demographic and health surveys of nine East African countries reported a similar finding (17). The finding implies that rural women need extra vigilant attention in providing maternal health services, particularly family planning that can alleviate important constructs of high-risk fertility such as birth spacing and birth order. It further pointed toward the need for embarking on the culture of early marriage in rural areas of Ethiopia as this is one of the factors that contributed to maternal high-risk fertility.

The probability of having high-risk fertility behavior was three times higher for mothers aged 14-19 years at marriage compared to those 20-25 years old at the time of marriage. This gives a chance to early reproduction leading to high birth order at later ages contributing to high-risk fertility and birth at an age less than 18 was also one of the constructs of maternal high-risk fertility behavior. Moreover, those mothers with the last unplanned but wanted pregnancy type were three times more likely to experience high-risk fertility compared to those whose last pregnancy type was planned and wanted. Another study that analyzed the Ethiopian DHS revealed that unwanted pregnancies were determinants of high-risk fertility behavior (13). Women who had histories of unwanted pregnancies were more likely to have high-risk fertility behavior than wanted pregnancies as per the findings from Ethiopia and Nigeria (2, 14). The explanation behind this factor might be the reason that women who may not utilize family planning methods could face unplanned and unwanted pregnancies, and this would contribute to high-risk fertility as a result of too close birth spacing and high birth orders.

The findings of this study may have limitations due to its focus on hospital-based births, which could result in an overestimation of high-risk fertility rates. Additionally, the results of the study may not apply to women at private hospitals in the area, as the research was only conducted in public hospitals.

This study found that a significant percentage of married women in the study area engaged in high-risk fertility behavior. Mothers who lived in rural areas, had early marriages, and unplanned last pregnancies were more likely to be engaged in high-risk fertility patterns. Plans for interventions aimed at preventing maternal high-risk fertility behavior should center on expanding access to family planning services and ending the practice of early marriage by giving rural women extra care and attention.
